# Trabectedin in the Neoadjuvant Treatment of High-Grade Pleomorphic Sarcoma: Report of a Rare Case and Literature Review

**DOI:** 10.1155/2013/320797

**Published:** 2013-01-16

**Authors:** Markus K. Schuler, Stephan Richter, Ivan Platzek, Bettina Beuthien-Baumann, Kathrin Wieczorek, Christine Hamann, Johannes Mohm, Gerhard Ehninger

**Affiliations:** ^1^Department of Internal Medicine I, University Hospital Carl Gustav Carus, Dresden University of Technology, 01307 Dresden, Germany; ^2^Department of Radiology, University Hospital Carl Gustav Carus, Dresden University of Technology, 01307 Dresden, Germany; ^3^Department of Nuclear Medicine, University Hospital Carl Gustav Carus, Dresden University of Technology, 01307 Dresden, Germany; ^4^Department of Pathology, University Hospital Carl Gustav Carus, Dresden University of Technology, 01307 Dresden, Germany; ^5^Department of Orthopaedics, University Hospital Carl Gustav Carus, Dresden University of Technology, 01307 Dresden, Germany; ^6^Hämatologisch-Onkologische Praxis, Dresden, Germany

## Abstract

*Background*. Pleomorphic sarcoma is an aggressive soft tissue sarcoma. In patients with high-risk extremity sarcomas, the significant survival benefits conferred by an intense regimen of neoadjuvant chemoradiotherapy and surgery were reported. To our knowledge, this is the first report in the literature of the neoadjuvant use of trabectedin in a patient with high-grade pleomorphic sarcoma, ineligible for standard neoadjuvant combination therapy with an anthracycline-based regimen. *Case Presentation*. Here we present a 58-year-old White male with a large tumor in the left thigh, but with no signs of metastases. Owing to the history of severe heart attack, three cycles of neoadjuvant trabectedin were administrated to achieve surgically wide margins. After two cycles, an 18F-FDG-PET showed a large proportion of the central tumor area was without metabolic activity. According to RECIST and Choi criteria, the tumor was stable. After the third cycle of trabectedin, the patient underwent a complete resection, which revealed completely necrotic high-grade pleomorphic sarcoma (stage pT2b), with only a small vital area. *Conclusion*. The present paper on a promising treatment with neoadjuvant trabectedin of patients with high-grade pleomorphic sarcoma might suggest that such treatment approach may provide a greater chance of cure and survival of such patients.

## 1. Background

The high-risk stage III soft tissue sarcomas (STS) are a heterogeneous group of relatively rare mesenchymal neoplasms with important differences in chemosensitivity. The use of chemotherapy in the neoadjuvant and adjuvant settings in STS is controversial. When preoperative chemotherapy is used, tumor responses can result in clinically meaningful tumor shrinkage prior to surgery and, thus, may facilitate subsequent surgical resection. Nowadays there are some pieces of evidence for the use of perioperative chemotherapy in this highly malignant disease. Mullen and colleagues have reported the significant survival benefits in patients with high-risk, extremity STS who received an intense regimen of neoadjuvant chemoradiotherapy with concomitant radiotherapy [1]. Another prospective phase II trial by Kraybill et al. demonstrated a comparably good outcome with an overall survival of 71% after 5 years in patients with high-grade, high-risk STS treated with neoadjuvant chemotherapy, preoperative radiotherapy, and adjuvant chemotherapy [2]. Meta-analysis found a significant advantage in relapse-free and overall survival. Nevertheless, in light of missing prospective randomized trials showing a reproducible benefit it is still a matter of debate which patients will have a benefit from this approach. Besides, apart from certain entities, such as myxoid liposarcoma, no histology-driven trials have yet been performed [3, 4]. Therefore, there is still lacking evidence for the use of chemotherapy in the neoadjuvant setting in patients with STS.

Treatment guidelines of sarcoma research and treatment organizations so far recommend a shared decision-making process considering among others patients age, tumor-subtype, grading, and size [5]. According to a French analysis especially patients with grade 3 tumors might benefit from adjuvant chemotherapy [6].

To date, the standard for perioperative chemotherapy is combining anthracycline with high-dose ifosfamide [5, 7]. This regimen is quite toxic and not applicable to a significant proportion of elderly and frail people. Therefore, these patients cannot be treated in a perioperative way, since less toxic regimens have not yet been evaluated. 

Trabectedin (Yondelis) is a marine-derived antineoplastic compound isolated from the Caribbean tunicate *Ecteinascidia turbinata* and currently produced synthetically [8]. Trabectedin is approved in the European Union and many other countries worldwide for the treatment of advanced STS after the failure of anthracyclines and ifosfamide or for patients who are unsuited to receive these agents. It has a unique mechanism of action based on interaction with the minor groove of the DNA double helix, which triggers a cascade of events that interfere with several transcription factors, DNA binding proteins and DNA repair pathways, resulting in G2-M cell cycle arrest and ultimately apoptosis [9]. Moreover, trabectedin at therapeutic concentrations has selective anti-inflammatory and immunomodulatory properties on tumor microenvironment as it decreases the production of factors potentially relevant for tumor growth, progression, angiogenesis, and metastasization. In particular, the *in vitro* production of proinflammatory mediators, such as interleukin-6 (IL-6) and CCL2, was markedly downregulated by trabectedin in circulating monocytes, macrophages, and tumor-associated macrophages (TAM) from human tumors [9–11]. Therefore, the mechanism of action of trabectedin has dual effect: it acts not only on tumor growth but also on tumor immune inflammation and angiogenesis, providing a consolidated therapeutic approach as a multitarget drug. 

Trabectedin shows nearly similar response rates in leiomyosarcoma and liposarcomas (L-sarcomas) as those in combination-therapies and by far is less toxic [12]. In addition, trabectedin was generally well tolerated and showed manageable toxicity when administered to patients with myxoid liposarcoma as neoadjuvant chemotherapy [3]. Based on these findings, a neoadjuvant treatment strategy with trabectedin could be extremely beneficial for those patients, especially those who are not eligible for an aggressive combination therapy. Apart from its potential local and systemic benefit, trabectedin treatment prior to surgery could result in a higher completion rate of planned chemotherapy cycles. Morbidity after local resection can often hinder the application of systemic therapy, consecutively leading to an undertreatment of high-risk patients.

Herein we present the results of the neoadjuvant use of trabectedin in a patient with high-grade pleomorphic sarcoma, ineligible for standard neoadjuvant combination therapy with an anthracycline-based regimen.

## 2. Case Report

A 58-year-old White male presented with a large tumor in the left thigh. The patient had a history of severe cardiac problems with myocardial infarction two years before diagnosis and consecutively underwent prophylactic defibrillator implantation, having an ejection fraction of 45% after surgery. A cross-sectional imaging showed an approximately 30 × 14 × 11 cm lobulated tumor. Staging was completed with a computerized tomography (CT) scan of the chest and abdomen and a fluorine-18 fluorodeoxyglucose (FDG)-positron emission tomography (PET) scan. This scan confirmed the presence of the mass and revealed an increased FDG uptake with a maximal standardized uptake value of 10.5 in the proximal region. No metastases were detected. Incision biopsy finally confirmed a high-grade sarcoma without distinct classification. Microscopic findings discovered up to 10 mitoses in 10 high-power fields (HPF) and a 30% necrosis.

**Figure 1:**
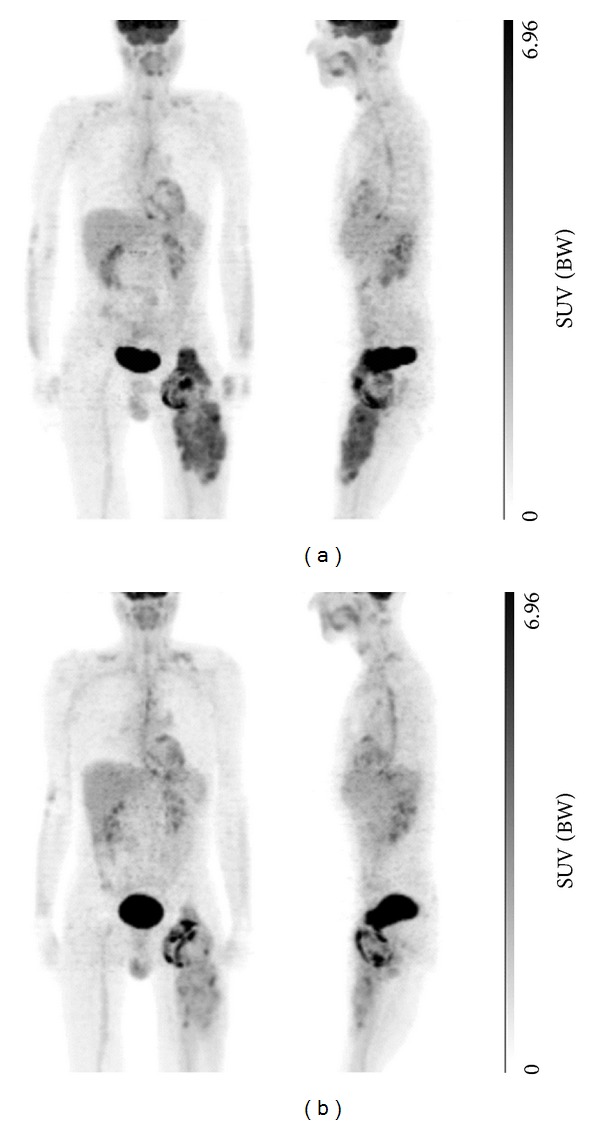
18F-FDG-PET maximum-intensity-projections (MIPS), (a) before the start of CTx and (b) after 2 cycles of CTx.

**Figure 2:**
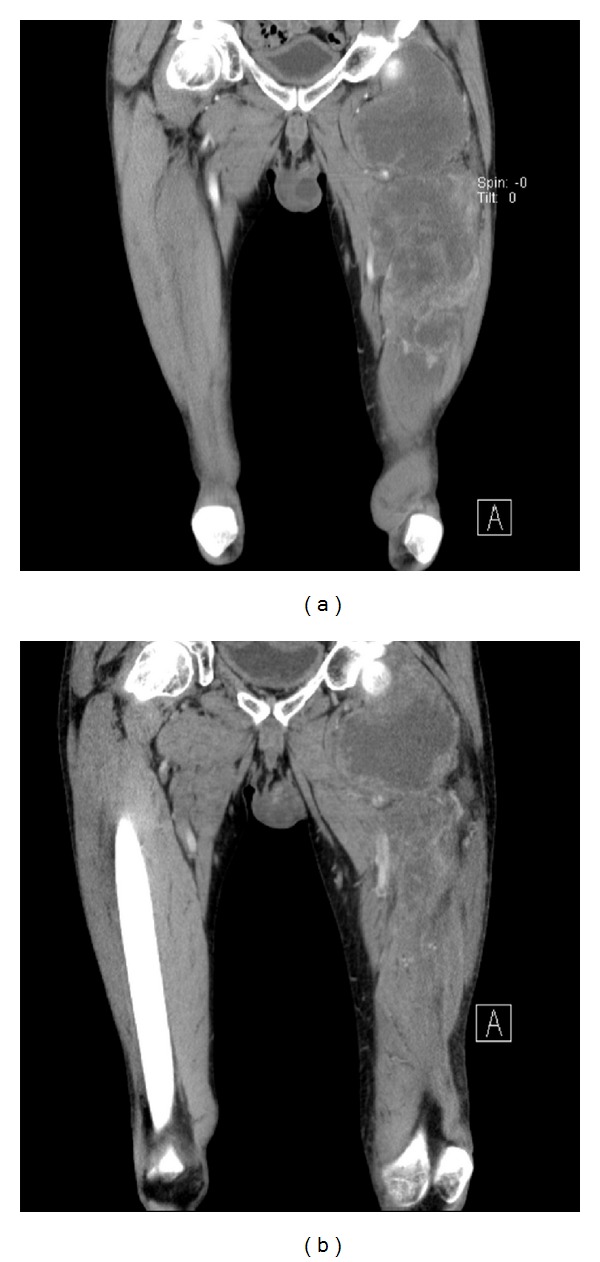
CT scan, (a) before the start of CTx and (b) after 2 cycles of CTx.

 Neoadjuvant chemotherapy was recommended in order to achieve surgically wide margins. Because of the history of severe heart attack, the patient was unsuited to receive anthracycline-based chemotherapy. As an individual approach with the patient, trabectedin was chosen for the patient-tailored treatment. Three cycles of trabectedin were given on day 1 every 3 weeks at the dose of 1.5 mg/m^2^ body surface area, administered as an intravenous infusion over 24 hours. After two cycles, an 18F-FDG-PET was performed, showing no significant decrease in standardized uptake value (SUV) in the hyper-metabolic regions of the tumor: maximal SUV was 9.5 *versus* 10.5 in the proximal and 4.4 *versus* 5.2 in the distal tumor (Figure 1). However, a large proportion of the central tumor area showed no metabolic activity. When applying the response evaluation criteria in solid tumors (RECIST) and the Choi criteria (Figure 2) to the CT scan, the tumor was stable. An analysis of tumor volumetric revealed a 35% decrease (from 956 cm³ to 621 cm³) of the tumor mass.

**Figure 3:**
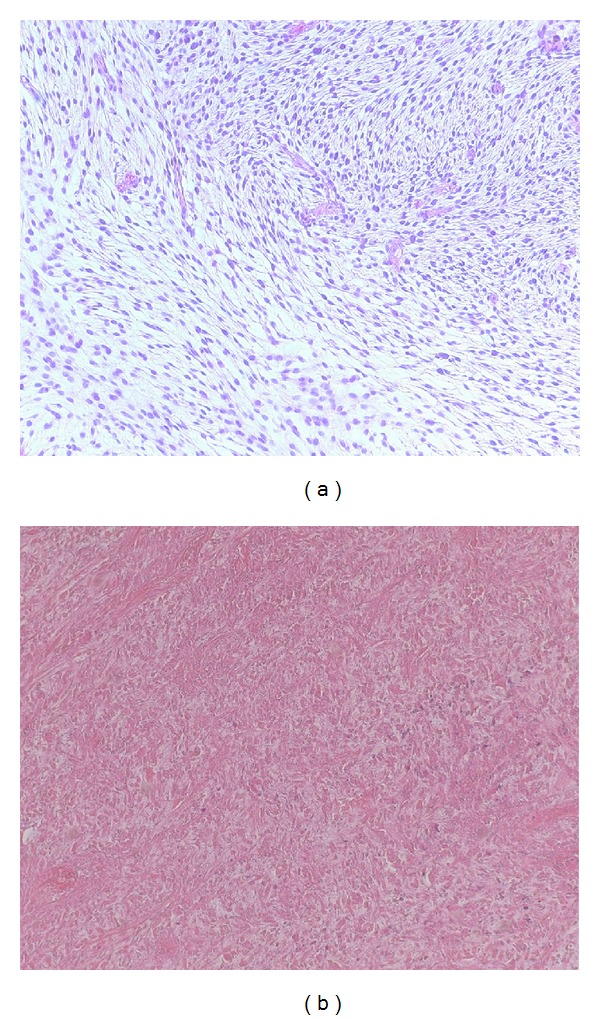
(a) Densely cellular neoplasm composed of round- and spindle-shaped cells.; (b) necrosis and hyalinization after neoadjuvant therapy, (H&E staining, 10x).

 After three cycles of trabectedin the patient was referred to resection. The postoperative classification after complete resection of the femur was stage pT2b high-grade pleomorphic sarcoma with 0.5 cm negative margins towards the acetabulum. The only vital tumor area was in the dorso-cranial area with viability of 15%, while all other parts showed a complete necrosis (Figure 3).

## 3. Literature Review 

In a former phase II clinical trial three of 23 patients with myxoid liposarcoma treated with neoadjuvant trabectedin achieved a pathological complete response (13%; 95% 95% confidence interval [CI]: 3%–34%) and another 12 of 23 had at least a good regression rate (>50% regression) as assessed by central pathological review [3]. In all the regressed cases, the decrease of cellular components paralleled the replacement with an acellular stromal component. Therefore, earlier studies using trabectedin in advanced disease might have underestimated the benefit of this drug on tumor devitalisation when using conventional measurement for the assessment of tumor response [13–15].

 After only three cycles of the recommended dose of trabectedin (1.5 mg/m^2^ given intravenously over 24 hours every 21 days) the tumor showed a very good pathological response with 85% of necrotic area. Given that histopathological response can translate into a better overall response, this represents a promising and meaningful finding [16]. Since prospective data about pathological response in sarcoma other than myxoid liposarcoma are missing, our results in this non-L-type sarcoma may help to better correlate different imaging techniques with the biology of the tumor. The fact that upon simultaneous functional and cross-sectional imaging there was only a minor response again raises the question, whether the use of RECIST criteria will be the adequate marker of response. Recently, it has been published that Choi criteria much better match with overall survival than RECIST criteria in patients undergoing preoperative chemotherapy [17].

The duration of therapy was chosen in concordance to the publication of Gronchi et al., showing that three cycles of neoadjuvant chemotherapy are equal to five cycles administered perioperatively [3]. Today it remains unknown, whether three cycles of single drug trabectedin are equal to three cycles of combination therapy with anthracycline and ifosfamide in the neoadjuvant setting. Furthermore, one must question the appropriate duration of neoadjuvant trabectedin. When Gronchi and colleagues compared their work with the results of trabectedin used in the same histology in palliative treatment, they found that differences in objective response by RECIST criteria could be partially explained by differences in treatment duration between the neoadjuvant and the advanced therapy. For instance, neoadjuvant treatment was limited in the study of Gronchi et al. to a maximum of six cycles, while in the study by Grosso et al. patients received a median number of 10 cycles in the advanced setting [18, 19]. Late responses are often observed with trabectedin; therefore, our patient might also have experienced a further remission after continued treatment [20].

## 4. Conclusions

In conclusion, this is the first report on a promising treatment with neoadjuvant trabectedin of high-grade pleomorphic sarcoma. More research is needed, particularly regarding the selection of patients who would benefit the most from appropriate neoadjuvant perioperative chemotherapy.

## References

[1] Mullen JT, Kobayashi W, Wang JJ (2012). Long-term follow-up of patients treated with neoadjuvant chemotherapy and radiotherapy for large, extremity soft tissue sarcomas. *Cancer*.

[2] Kraybill WG, Harris J, Spiro IJ (2010). Long-term results of a phase 2 study of neoadjuvant chemotherapy and radiotherapy in the management of high-risk, high-grade, soft tissue sarcomas of the extremities and body wall: Radiation Therapy Oncology Group Trial 9514. *Cancer*.

[3] Gronchi A, Bui BN, Bonvalot S (2012). Phase II clinical trial of neoadjuvant trabectedin in patients with advanced localized myxoid liposarcoma. *Annals of Oncology*.

[4] Casali PG (2012). Histology- and non-histology-driven therapy for treatment of soft tissue sarcomas. *Annals of Oncology*.

[5] Casali PG, Blay JY (2010). Soft tissue sarcomas: ESMO clinical practice guidelines for diagnosis, treatment and follow-up. *Annals of Oncology*.

[6] Italiano A, Delva F, Mathoulin-Pelissier S (2010). Effect of adjuvant chemotherapy on survival in FNCLCC grade 3 soft tissue sarcomas: a multivariate analysis of the French Sarcoma Group Database. *Annals of Oncology*.

[7] Penel N, Van Glabbeke M, Marreaud S, Ouali M, Blay JY, Hohenberger P (2011). Testing new regimens in patients with advanced soft tissue sarcoma: analysis of publications from the last 10 years. *Annals of Oncology*.

[8] Carter NJ, Keam SJ (2010). Trabectedin: a review of its use in soft tissue sarcoma and ovarian cancer. *Drugs*.

[9] D’Incalci M, Galmarini CM (2010). A review of trabectedin (ET-743): a unique mechanism of action. *Molecular Cancer Therapeutics*.

[10] Allavena P, Signorelli M, Chieppa M (2005). Anti-inflammatory properties of the novel antitumor agent yondelis (trabectedin): inhibition of macrophage differentiation and cytokine production. *Cancer Research*.

[11] Germano G, Frapolli R, Simone M (2010). Antitumor and anti-inflammatory effects of trabectedin on human myxoid liposarcoma cells. *Cancer Research*.

[12] Demetri GD, Chawla SP, von Mehren M (2009). Efficacy and safety of trabectedin in patients with advanced or metastatic liposarcoma or leiomyosarcoma after failure of prior anthracyclines and ifosfamide: results of a randomized phase II study of two different schedules. *Journal of Clinical Oncology*.

[13] Garcia-Carbonero R, Supko JG, Maki RG (2005). Ecteinascidin-743 (ET-743) for chemotherapy-naive patients with advanced soft tissue sarcomas: multicenter phase II and pharmacokinetic study. *Journal of Clinical Oncology*.

[14] Yovine A, Riofrio M, Blay JY (2004). Phase II study of ecteinascidin-743 in advanced pretreated soft tissue sarcoma patients. *Journal of Clinical Oncology*.

[15] Le Cesne A, Blay JY, Judson I (2005). Phase II study of ET-743 in advanced soft tissue sarcomas: a European Organisation for the Research and Treatment of Cancer (EORTC) soft tissue and bone sarcoma group trial. *Journal of Clinical Oncology*.

[16] Herrmann K, Benz MR, Czernin J (2012). 18F-FDG-PET/CT Imaging as an early survival predictor in patients with primary high-grade soft tissue sarcomas undergoing neoadjuvant therapy. *Clinical Cancer Research*.

[17] Stacchiotti S, Verderio P, Messina A (2012). Tumor response assessment by modified Choi criteria in localized high-risk soft tissue sarcoma treated with chemotherapy. *Cancer*.

[18] Grosso F, Jones RL, Demetri GD (2007). Efficacy of trabectedin (ecteinascidin-743) in advanced pretreated myxoid liposarcomas: a retrospective study. *Lancet Oncology*.

[19] Grosso F, Sanfilippo R, Virdis E (2009). Trabectedin in myxoid liposarcomas (MLS): a long-term analysis of a single-institution series. *Annals of Oncology*.

[20] Casali PG, Sanfilippo R, D’Incalci M (2010). Trabectedin therapy for sarcomas. *Current Opinion in Oncology*.

